# Use of tiotropium bromide in an adolescent with adrenal suppression secondary to inhaled mometasone furoate

**DOI:** 10.1186/1710-1492-10-S2-A14

**Published:** 2014-12-18

**Authors:** Mariam Hanna, Douglas P Mack

**Affiliations:** 1 Dept of Clinical Immunology and Allergy, McMaster University, Hamilton, ON, Canada; 2 Dept of Pediatrics, McMaster University, Hamilton, ON, Canada

## Introduction

With emerging cases of adrenal suppression being reported in pediatric patients, finding appropriate steroid sparing agents is of clinical importance. Tiotropium bromide (TB), a long-acting anticholinergic, has recently been investigated in adults as a steroid-sparing agent in asthma step-up therapy. We report the first case of tiotropium bromide (TB) used as a step-up steroid sparing agent in an adolescent with adrenal suppression secondary to inhaled mometasone furoate (MF).

## Case description

A sixteen-year-old female with asthma and allergic rhinitis treated MF/formoterol fumarate 800/ 20 mcg per day in addition to intranasal MF presented with increasing fatigue, weight loss, nausea and striae. Extensive initial hospital investigations were normal. An AM cortisol was reported < 50 nmol/L (170-540). She was started on hydrocortisone therapy and changed to ciclesonide 400 mcg, as well as montelukast and formoterol. Her energy and weight improved after initiation of replacement hydrocortisone therapy. Despite using sublingual grass immunotherapy, during grass pollen season the patient's asthma control worsened. Rather than increase the dose of inhaled corticosteroids, 18 mcg of daily TB was added for a therapeutic trial. The patient experienced symptomatic improvement with less nocturnal cough, normal exercise tolerance and 4% improvement in FEV1.

## Discussion

There is a limited repertoire of steroid sparing agents available for use in the pediatric asthma population. While adrenal suppression due to inhaled corticosteroids is considered relatively uncommon, and prior MF-induced adrenal suppression has only been reported by the authors, finding suitable alternatives to inhaled corticosteroids is paramount in this population. Physicians may consider a trial of TB in an attempt to decrease the dose of inhaled corticosteroids in their pediatric patients to avoid adrenal suppression.

## Consent

Written informed consent was obtained from the patient for publication of this abstract and any accompanying images. A copy of the written consent is available for review by the Editor of this journal.

